# Plasmonic Manipulation of DNA using a Combination of Optical and Thermophoretic Forces: Separation of Different-Sized DNA from Mixture Solution

**DOI:** 10.1038/s41598-020-60165-5

**Published:** 2020-02-25

**Authors:** Tatsuya Shoji, Kenta Itoh, Junki Saitoh, Noboru Kitamura, Takahiro Yoshii, Kei Murakoshi, Yuto Yamada, Tomohiro Yokoyama, Hajime Ishihara, Yasuyuki Tsuboi

**Affiliations:** 1 Division of Molecular Materials Science, Graduate School of Science, Osaka City University, 3-3-138 Sugimoto, Sumiyoshi, Osaka 5558-8585 Japan; 2 The OCU Advanced Research Institute for Natural Science and Technology (OCARINA), Osaka City University, 3-3-138 Sugimoto, Sumiyoshi, Osaka 5558-8585 Japan; 3 Department of Chemistry, Graduate School of Science, Hokkaido University, Sapporo, Hokkaido 060-0810 Japan; 4 Division of Materials Physics, Graduate School of Engineering Science, Osaka University, 1-3 Machikaneyama, Toyonaka, Osaka 560-8531 Japan; 5 Department of Physics and Electronics, Graduate School of Engineering, Osaka Prefecture University, 1-1, Gakuen-cho, Nakaku, Sakai, Osaka 599-8531 Japan

**Keywords:** Physical chemistry, Optical manipulation and tweezers

## Abstract

We demonstrate the size-dependent separation and permanent immobilization of DNA on plasmonic substrates by means of plasmonic optical tweezers. We found that a gold nanopyramidal dimer array enhanced the optical force exerted on the DNA, leading to permanent immobilization of the DNA on the plasmonic substrate. The immobilization was realized by a combination of the plasmon-enhanced optical force and the thermophoretic force induced by a photothermal effect of the plasmons. In this study, we applied this phenomenon to the separation and fixation of size-different DNA. During plasmon excitation, DNA strands of different sizes became permanently immobilized on the plasmonic substrate forming micro-rings of DNA. The diameter of the ring was larger for longer DNA (in base pairs). When we used plasmonic optical tweezers to trap DNA of two different lengths dissolved in solution (φx DNA (5.4 kbp) and λ-DNA (48.5 kbp), or φx DNA and T4 DNA (166 kbp)), the DNA were immobilized, creating a double micro-ring pattern. The DNA were optically separated and immobilized in the double ring, with the shorter sized DNA and the larger one forming the smaller and larger rings, respectively. This phenomenon can be quantitatively explained as being due to a combination of the plasmon-enhanced optical force and the thermophoretic force. Our plasmonic optical tweezers open up a new avenue for the separation and immobilization of DNA, foreshadowing the emergence of optical separation and fixation of biomolecules such as proteins and other ncuelic acids.

## Introduction

DNA has been the main target for optical trapping and manipulation. This is very important in medical science and biological physics, and numerous studies have been carried out so far. With conventional optical tweezers simply using a focused laser beam (developed by Arthur Ashkin^1–4^), it is rather difficult to optically trap DNA in aqueous solutions containing hydrated random coil structures because of their small polarizability^5^. In many cases, small dielectric microspheres are connected to the head and end groups of the DNA, and a focused laser beam optically manipulates these microspheres^6–9^. This technique for DNA manipulation has stimulated extensive development in DNA biophysics.

Recently, a breakthrough was achieved in the research field of optical tweezers^10–15^. This is based on surface plasmons localized around metallic nanostructures. The surface plasmons lead to an electric field (***E***) enhancement effect of the incident light which amplifies the optical force (***F***_*g*_, gradient force) and hence the optical trapping potential (*U*):

1 $${{\boldsymbol{F}}}_{{\bf{g}}}=-\frac{1}{2}\alpha \nabla {{\boldsymbol{E}}}^{2}$$

2 $$U=\frac{1}{2}\alpha {E}^{2}$$

where *α* is the polarizability of the micro/nano-particle to be trapped. In the vicinity of the metallic nanostructure (in many cases it is a nanogap), ***E*** is highly localized around the nanogap, resulting in a huge enhancement in ∇***E***^2^. Therefore, the “grip” exerted on nanomaterials by plasmonic optical tweezers or plasmonic optical trapping (POT) should be very strong^16–18^. Following early demonstrations, POT has undergone rapid growth, and has been used to trap various nanomaterials such as polymer beads^10,19–27^, metallic nanoparticles^12,18^, quantum dots^13,28,29^, dye aggregates^30,31^, and so on. In addition to these hard nanospheres, we have demonstrated that POT is also applicable to soft materials such as flexible polymer chains homogeneously dissolved in water^32,33^. In particular, we succeeded in POT of lambda DNA (48.5 kbp, abbreviated as λ-DNA) without microbeads, as described below^34^. Gordon also succeeded in POT of a single protein molecule using a double-hole nanostructure in a thin gold film^35^.

In the early stages of these studies, POT has been used simply to trap and release these small objects. Researchers have observed trapping of particles upon plasmon excitation under an optical microscope. Following these studies, some researchers demonstrated arranging or patterning particles using self-organization effects or physical external perturbation. In 2013, we demonstrated that POT of λ-DNA using a continuous-wave (CW) laser beam (λ = 808 nm) could be used to permanently fix DNA onto a plasmonic substrate^34^. On the other hand, when we replaced the CW laser by a femtosecond pulsed laser, we were able to drive the POT in a simple trap-and-release mode. Beyond such *static* POT, Tsai *et al*. demonstrated rotation of a microparticle by irradiating a chiral spiral structure on a metallic film with circularly polarized light^36^.

These studies strongly indicate that POT can be extended to the manipulation of a variety of nano-materials. On the other hand, it has frequently been pointed out that POT is accompanied by a local rise in temperature, because POT involves excitation of the free electrons in a metal, leading to heat generated by electron-lattice energy relaxation. Such local rises in temperature frequently create huge temperature gradients, possibly affecting the POT behavior via thermophoresis^37–41^. In most cases thermophoresis takes a particle from a hotter region (central area of an illumination spot) to a colder region (outer area of the spot). This means that thermophoresis acts as a dispersive force, hindering the stability of POT. POT is susceptible to thermophoresis and significant efforts have been made to understand and suppress such rises in local temperature^42–44^. To clarify and get a good understanding of POT, we have quantitatively evaluated the local rise in temperature (*ΔT*) and the thermophoretic force (***f***_*t*_) in POT for a gold nano-pyramidal dimer array by means of fluorescence correlation spectroscopy, and revealed that the thermophoretic force is never negligible and can significantly compete with the plasmon-enhanced optical force^32^. Such thermal forces presumably limit the future development of POT.

In our previous work on POT of λ-DNA, the DNA molecules immobilized on the plasmonic substrate created a ring shaped DNA assembly^34^. We consider that the DNA ring originates from a balance between the plasmon-enhanced optical force and the thermophoretic force. This suggests that coupling POT with thermal effects can open up a new area involving the novel manipulation of small objects. In this study, we apply this phenomenon to the separation and trapping of DNA. POT was performed on solutions containing a mixture of large and small DNA with different color fluorescence. Based on the fact that the optical and thermophoretic forces are sensitive to particle size, we examined the possibility of optical separation and fixation of two different-sized DNA from mixture solution (5.4 kbp DNA and λ-DNA, or 5.4 kbp DNA and 166 kbp DNA).

## Results

**Figure 1:**
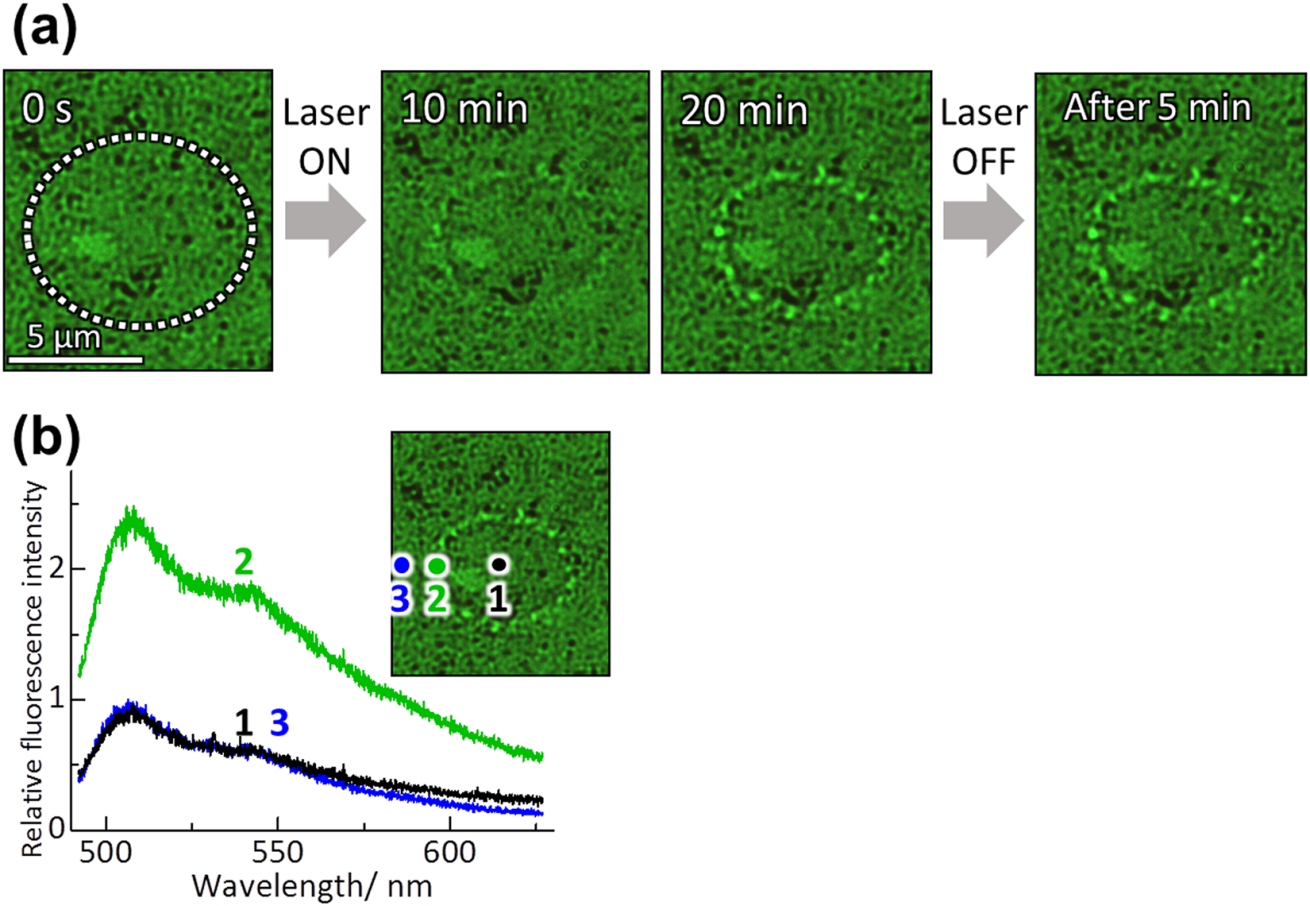
(**a**) Bright-field micrographs of plasmonic optical trapping (POT) of λ-DNA (2.0 × 10^−6^ mol/*l*) stained with YOYO-1. The area irradiated by the laser (λ = 808 nm, *I* = 15 kW/cm^2^) is shown as a circle of white dots. (**b**) Spatially-resolved fluorescence spectra for the DNA micro-ring: (black, 1) at the center of the irradiated area, (green, 2) just at the ring, (blue, 3) outside the ring.

Irradiating a sample solution in contact with the plasmonic nanostructure (polarized extinction spectra and SEM images are shown in Fig. S1) with the 808 nm laser beam (Optical setup is shown in Fig. S2) resulted in trapping of the DNA. We used three different-sized DNA with a swollen random coil configurations in buffer solution (see in Methods section): φx174 DNA (5.4 kbp, abbreviated as φx DNA), lambda DNA (48.5 kbp, abbreviated as λ-DNA), and T4 GT7 DNA (165.6 kbp, abbreviated as T4 DNA). For the solution, we carried out dynamic light scattering measurements to determine hydrodynamic diameter of these DNA: 240 nm (φx DNA), 330 nm (λ-DNA), and 760 nm (T4 DNA). As an example, the POT behavior of λ-DNA (48.5 kbp) stained with YOYO-1 is described in detail here. Note here that POT of λ-DNA has been reported in elsewhere by us^34^. Figure 1(a) displays a series of micrographs for POT of DNA with an excitation intensity *I* = 15 kW/cm^2^.

As can clearly be seen in these micrographs, a micro-ring was formed in the irradiated area (circle of white dots). Figure 1(b) shows spatially-resolved fluorescence spectra for the ring. At the center of the irradiated area and outside the ring, we slightly observed background fluorescence from DNA dispersed in solution (2.0 × 10^−6^ mol/L). On the other hand, from the ring, fluorescence from YOYO-1 is clearly evident. This indicates that the ring is formed of λ-DNA. Thus, a ring-shaped assembly of λ-DNA has been formed by POT. Assuming that the fluorescence intensity was proportional to the amount of DNA, the concentration of DNA was 2.5 times higher than that of the solution. It should be noted that it is rough estimation because fluorescence quenching and enhancement by plasmon possibly take place competing each other. In the micro-ring, DNA would take random coil configurations and be entangled with each other because coil-to-globule transition of DNA hardly occurred in our experimental condition without cationic surfactants^45,46^. This result also indicates an important fact: a large part of the double-stranded (ds) DNA coils have been optically trapped without denaturation. It is noted that the elevated local temperature *T* = 61 °C of the irradiation area during plasmon excitation (see Discussion part) was lower than melting point of λ-DNA (99 °C)^47^. A photothermal effect by plasmon hardly induces thermal denaturation of the fixed DNA. In the present study, it is rather difficult to deny the possibility of the denaturation in the rings completely. However, based on the experimental results of the bright fluorescence from the rings and precise temperature determination, we consider that a large part of DNAs in the rings is not denatured.

As previously reported, the λ-DNA was not released even after stopping the irradiation, and we have observed the permanent fixation of the ring over 60 minutes^34^. These strands of DNA were permanently fixed on the surface of the plasmonic substrate. This is presumably due to adsorption based on the Coulombic interaction between the DNA and the surface of the glass substrate. The mechanism creating these micro-rings is thermophoresis (Soret effect), which is due to the huge temperature gradient resulting from plasmonic excitation. This effect is quantitatively discussed in a later section. We found that the sizes of the micro-rings could be controlled by changing the intensity of the irradiation for plasmonic excitation. Micro-rings were formed at intensities in the range of 10–20 kW/cm^2^. The threshold intensity for ring formation was *I* = 10 kW/cm^2^. When *I* > 20 kW/cm^2^, instead of POT, a micro-bubble appeared in the irradiated area due to a local rise in temperature as a result of the photothermal effect (electron-lattice relaxation in gold). In the range of 10 < *I* < 20 kW/cm^2^, the diameter of the micro-ring increased from 7 to 10 μm with increasing *I*. The ring size could also be controlled by changing the size of the irradiated area (see Figs. S3 and S4 in the supporting information).

**Figure 2:**
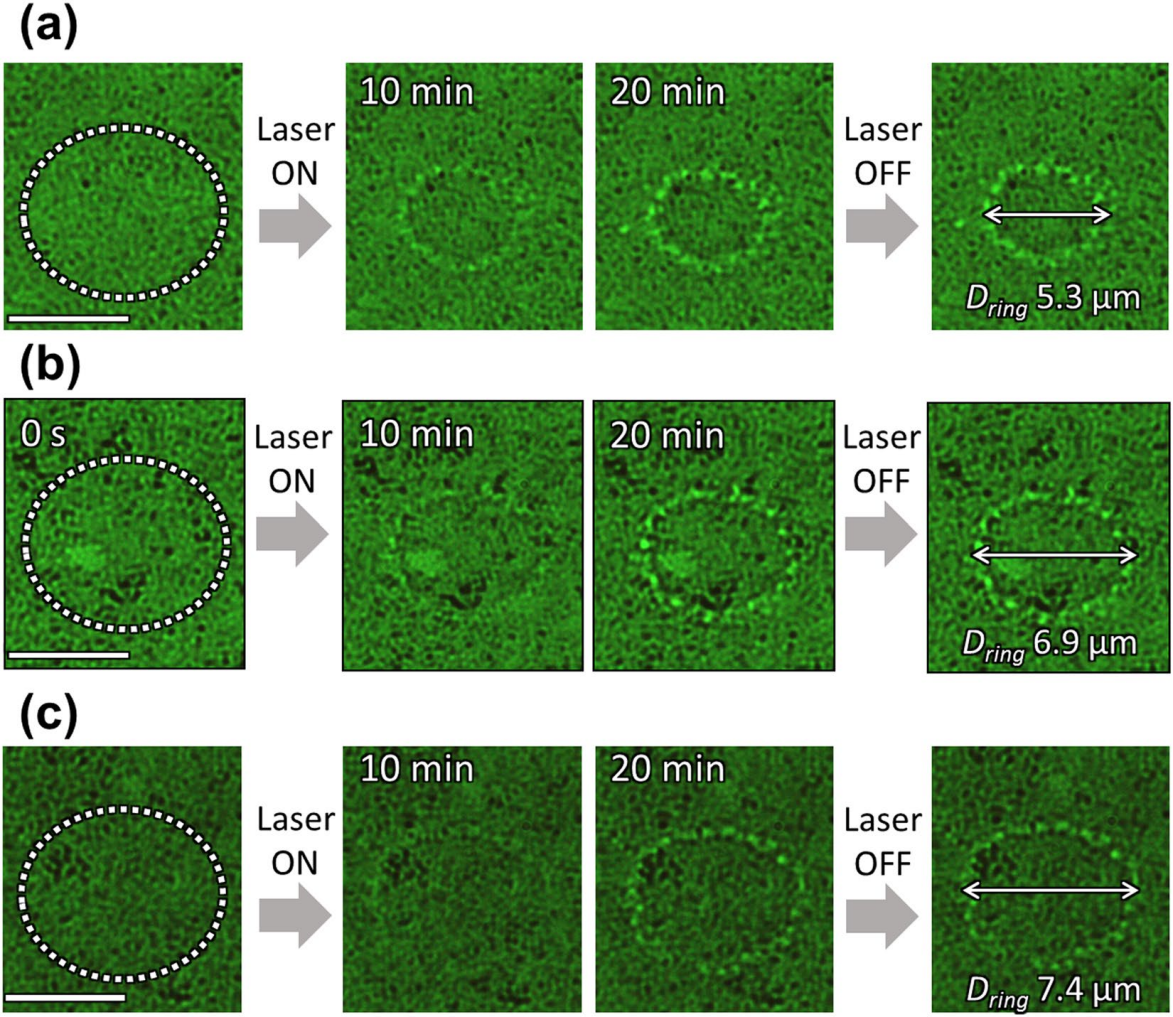
Bright-field micrographs of (**a**) φx DNA (5.4 kbp), (**b**) λ-DNA (48.5 kbp) (Fig. 1(a,c) T4 DNA (166 kbp). The DNA concentrations are 2.0 × 10^−7^ mol/*l*. The scale bars are 5 μm. The areas irradiated by the laser (λ = 808 nm, 15 kW/cm^2^) are shown by circles of white dots. Spatially-resolved fluorescence spectra of (**a**,**c**) are shown in the Supporting Information.

The formation of such micro-rings was also observed for the other types of DNA. What is important here is that the size of the rings in equilibrium (after 10 min. irradiation at 15 kW/cm^2^) clearly depends on the size of the DNA, as shown in Fig. 2. In the micrographs, it can clearly be seen that the size of the ring increases as the number of base pairs of DNA increases. In the figure, the diameters of the rings (*D*_*ring*_) are given for each number of base pairs of DNA; *D*_*ring*_ was evaluated to be 5.3 μm for 5.4 kbp DNA (φx DNA), 6.9 μm for 48.5 kbp DNA (λ-DNA) and 7.4 μm for 166 kbp (T4 DNA). This behavior is due to the thermophoretic force, which is sensitive to the size of microparticles and is discussed in a later section. It should be noted that we measured the fluorescence spectra of the rings in the same way as in Fig. 1, and identified each ring to consist of the corresponding DNA (see Fig. S4 in the supporting information).

**Figure 3:**
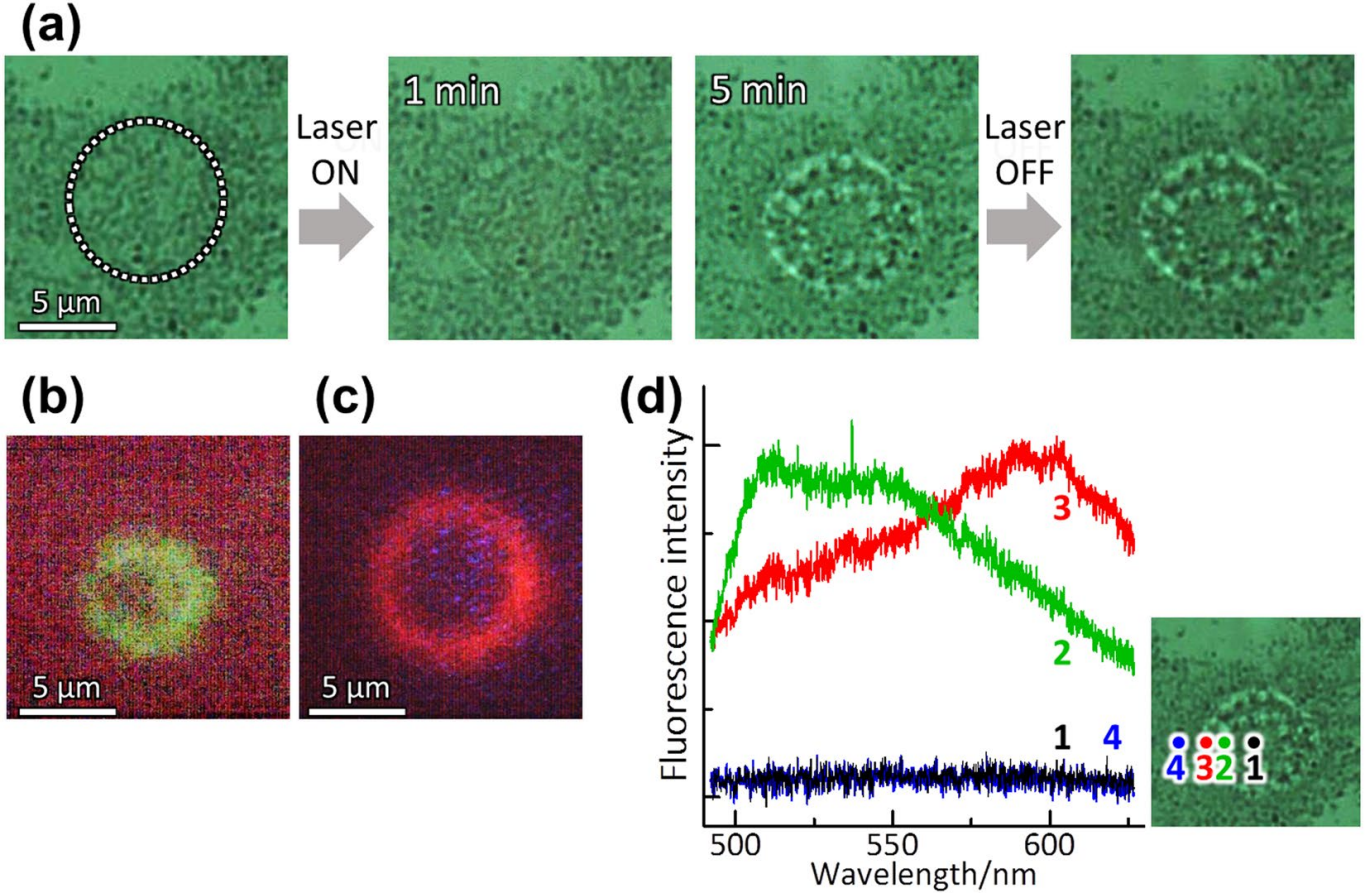
POT for a solution containing a mixture of φx DNA (5.4 kbp, 1.0 × 10^−6^ mol/*l*) labelled with YOYO-1 and T4 DNA(166 kbp, 1.0 × 10^−6^ mol/*l*) labelled with EtBr. (**a**) Bright-field micrographs. The area irradiated by the laser (λ = 808 nm, *I* = 10 kW/cm^2^) is shown by a circle of white dots. (**b**,**c**) Fluorescence micrographs obtained with different optical filters: (**b**) for green fluorescence from YOYO-1 and (**c**) for red fluorescence from EtBr. (**d**) Spatially-resolved fluorescence spectra: (black, 1) at the center of the irradiated area, (green, 2) just at the small inner micro-ring, (red, 3) just at the large micro-ring, and (blue, 4) outside of the double ring.

POT was carried out on solutions of mixed DNA, bearing in mind the possibility of applying this technique for separation and fixation of biomolecules such as proteins and other nucleic acids. Figure 3 shows the result of POT on a solution containing a mixture of φx DNA (5.4 kbp, 1.0 × 10^−6^ mol/*l*) labelled with YOYO-1and and T4 DNA(166 kbp, 1.0 × 10^−6^ mol/*l*) labelled with EtBr. The irradiation intensity was adjusted to be moderate (*I* = 10 kW/cm^2^). With irradiation, there was clear evidence of DNA trapping. Careful inspection of the micrographs (at 5.0 min. and after stopping the irradiation) revealed that concentric double rings (a large and a small ring) were produced and permanently immobilized in the irradiated area. Fluorescence images and fluorescence spectra are also shown in Fig. 3. By changing the optical filter in the microscope, we can clearly distinguish these two DNA rings via the color of the fluorescence (Fig. 3(b)). The observation of fluorescence emission supported that a large part of DNA was fixed without thermal denaturation, because the fluorescence dyes isolated from DNA hardly emitted in the solution. The large ring emits red fluorescence, while the small ring emits green fluorescence. This was also verified by spatially-resolved fluorescence microspectroscopy (Fig. 3(c)). Fluorescence from the large ring is identified as being from EtBr, while the fluorescence from the small ring is assigned to YOYO-1. Thus, these two kinds of DNA were clearly separated and immobilized by POT.

**Figure 4:**
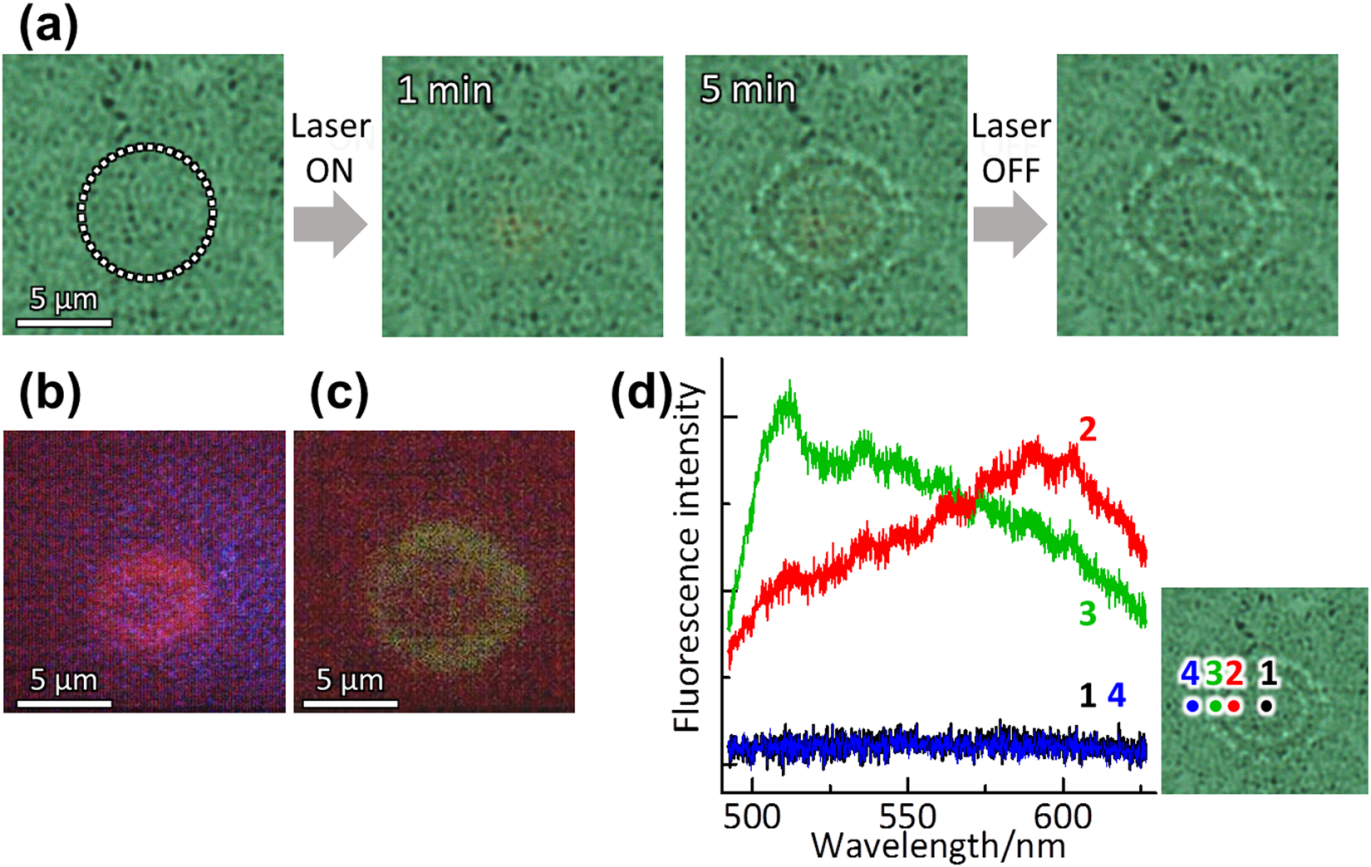
POT in a solution containing a mixture of φx DNA (5.4 kbp, 1.0 × 10^−6^ mol/L) labelled with EtBr and T4 DNA(166 kbp, 1.0 × 10^−6^ mol/L) labelled with YOYO-1. (**a**) Bright-field micrographs. The area irradiated with the laser (λ = 808 nm, *I* = 10 kW/cm^2^) is shown by a circle of white dots. (**b,c**) Fluorescence micrographs obtained with different optical filters: (**b**) for red fluorescence from EtBr and (**c**) for green fluorescence from YOYO-1. (**d**) Spatially-resolved fluorescence spectra: (black, 1) at the center of the irradiated area, (red, 2) just at the small inner micro-ring, (green, 3) just at the large micro-ring, and (blue, 4) outside the double ring.

A similar experiment with the fluorescent dyes exchanged was carried out on these DNA molecules. POT was examined for a solution containing a mixture of φx DNA labelled with EtBr and T4 DNA labelled with YOYO-1. Also in this case, as shown in Fig. 4, concentric double rings were produced and permanently immobilized in the irradiated area (Fig. 4(a)). The small ring emits the red EtBr fluorescence (Fig. 4(b), while the large ring emits green YOYO-1 fluorescence (Fig. 4(c). The fluorescence spectra for these two rings verify this. Again the result (Fig. 4) clearly indicates that these two kinds of DNA were optically trapped and immobilized on the plasmonic structure, and separated in accordance with their size.

**Figure 5:**
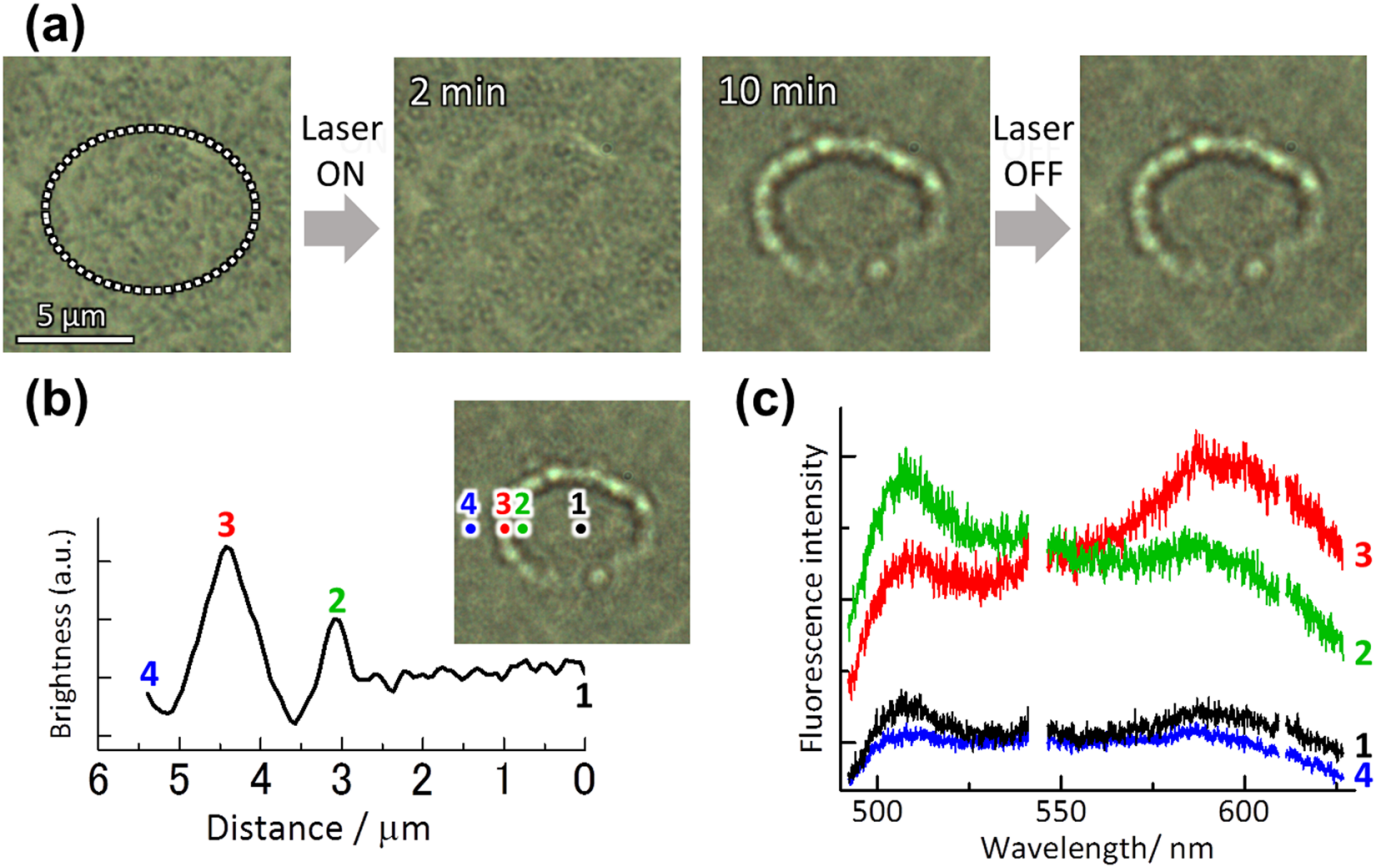
POT in a solution containing a mixture of φx DNA (5.4 kbp, 1.0 × 10^−6^ mol/*l*) labelled with YOYO-1 and λ-DNA(48.5 kbp, 1.0 × 10^−6^ mol/*l*) labelled with EtBr. (**a**) Bright-field micrographs. The area irradiated with the laser (λ = 808 nm, *I* = 15 kW/cm^2^) is shown by a circle of white dots. (**b**) Spatial profile (cross section) of brightness for the micrograph with the double micro-ring. (**c**) Spatially-resolved fluorescence spectra: (black, 1) at the center of the irradiated area, (green, 2) just at the small inner micro-ring, (red, 3) just at the large micro-ring, and (blue, 4) outside the double ring.

We also performed such trapping experiments on a solution containing a mixture of φx DNA (5.4 kbp, 1.0 × 10^−6^ mol/*l*) labelled with YOYO-1 and λ-DNA (48.5 kbp, 1.0 × 10^−6^ mol/*l*) labelled with EtBr. Figure 5(a) shows optical micrographs of such DNA trapping. A micro-ring appeared during plasmonic excitation for several minutes, eventually becoming a concentric double ring after irradiation for 10 minutes. Although the small inner ring is not very clear, the large outer ring can clearly be observed. Figure 5(b) shows a spatial profile (cross section) of the brightness of the micro-graph crossing the double ring. Both at the center (position 1) and outside the rings (position 4), the brightness is low. On the other hand, two peaks in the brightness were detected. Obviously, the high peak (position 3) and the low peak (position 2) correspond to the large and small rings, respectively. Since the small ring was not very clear, we measured the fluorescence spectrum at each position to identify the micro-rings. The results are shown in Fig. 5(c). Both at the center (position 1) and outside the rings (position 4), we only detected weak fluorescence signals, originating from the DNA dissolved in the sample solution (background fluorescence). These spectra are composed of a superposition of YOYO-1 and EtBr fluorescence.

In contrast, we clearly observed fluorescence from the micro-rings. As seen in the spectra, the large ring emits EtBr fluorescence (detected at position 3), while the small ring emits YOYO-1 fluorescence (detected at position 2). This means that the large ring consists of λ-DNA, while the small ring consists of φx DNA. In this way we succeeded in separating two kinds of DNA and patterning them into micro-rings by means of plasmonic optical tweezers.

## Discussion

In order to calculate the trapping force (gradient force) ***F***_*g*_, we have to know the value of *α* (the polarizability of the DNA) in Eq. (1). However, it is very difficult to precisely evaluate *α* for flexible DNA chains taking random coil structures in water. Umazano and Bertolotto theoretically calculated values of *α* (polarizability) for DNA at visible light frequencies^48^. They adopted the broken-rod macroion (BRM) model and applied the discrete dipole approximation (DDA), according to which each molecule is described as a finite array of electronic coupled oscillators. They estimated the value of *α* for model DNA in aqueous solution to be 2 × 10^−38^ F m^2^. We adopted this value in calculating ***F***_*g*_. Although it is an approximation, the *α* values of DNA do not change much even when the size of the hydrated DNA changes.

**Figure 6:**
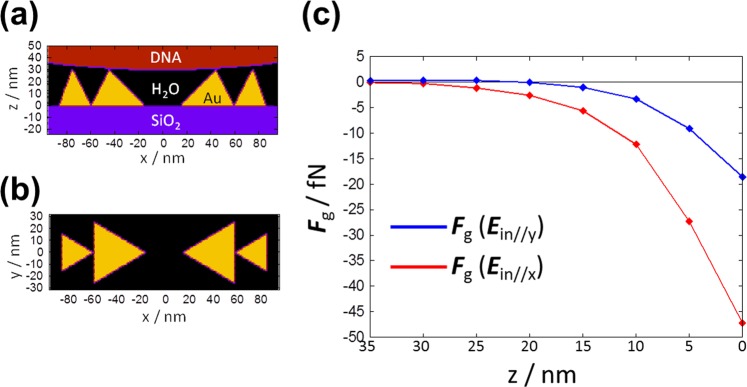
Theoretical calculation of the optical force exerted on the DNA around a plasmonic nano-pyramidal dimer. (**a**) Side and (**b**) top views of the dimer. (**c**) Calculated optical force exerted on the DNA in the *z*-direction: ***F***_g_(***E***_in//y_) is the electric field vector of the incident light (*I* = 15 kW/cm^2^) in the *y*-direction, while ***F***_g_(***E***_in//x_) is the vector in the *x*-direction.

The enhanced electromagnetic field (EMF) of the incident light around a gold nanopyramidal dimer was calculated using the discrete integral form of the Maxwell equations, which can be solved applying a discrete dipole approximation (DDA). Figure 6(a,b) show models of gold nanopyramids used for the calculations. The electromagnetic field of the incident light whose vector is parallel to the *x*-axis (***E***_in//x_) is considerably enhanced in the nanogaps between the nanopyramids with an enhancement factor (***E***^2^/***E***_0_^2^) reaching around 10^4^. The gradient of the electric field around the nanogaps (∇***E***^2^) can be obtained from such a calculation. In this way we can calculate the gradient force ***F***_*g*_ exerted on the DNA as a function of position along the *z*-axis. The results of the simulation are shown in Fig. 6(c), where ***F***_*g*_ when *I* = 15 kW/cm^2^ is plotted as a function of the distance between the DNA and the plasmonic nanostructure. When the hydrated DNA approaches the plasmonic structure (nano-pyramidal dimer), the value of |***F***_*g*_| increases from 0 (DNA just in contact with the top of the nanopyramids) to 50 fN (DNA covering the nanopyramids).

On the other hand, as we have demonstrated in a series of previous works, resonant light irradiation to the AR-NSL nanostructure results in plasmonic excitation, accompanied, simultaneously, by local heat generation^32,34^. This leads to a huge rise in temperature that drives the thermophoresis. Generally, and for DNA, thermophoresis causes transport from a hot region to a colder region^37,38^. Therefore, since the temperature in the plasmonic excitation area is higher than the surrounding areas, thermophoresis should act as a repulsive force, dispersing the DNA and hindering stable DNA trapping.

In addition to ***F***_g_, here we quantitatively evaluate the thermophoresis. The driving force for thermophoresis, also known as the Soret effect, is the thermophoretic force ***f***_*t*_, which is expressed as

3 $${f}_{t}=-\,{S}_{t}{k}_{B}T\nabla T$$

where *S*_t_ is the Soret coefficient, *T* is temperature, and ∇*T* is the temperature gradient^40^. The temperature *T* is

4 $$T={T}_{{\rm{room}}}+{\Delta }T$$

where *T*_room_ is room temperature (293 K) and *ΔT* is the rise in temperature by plasmonic excitation. Values of *S*_*t*_ for various types of DNA have been determined by Braun *et al*.^38,49^. In order to evaluate ***f***_*t*_, we measured the spatial temperature distribution around an area irradiated at *I* = 15 kW/cm^2^ (corresponding to the trapping experiments). In our previous studies, we evaluated the temperature by means of fluorescence correlation spectroscopy (FCS), which provided us with the diffusion coefficients (*D*) for a fluorescence probe in solvents^32,34^. The temperature was determined from the viscosity (*η*) of the solvent (*η* is a function of *T*). In the present study, we adopted a simpler method. We precisely evaluated *T* using a fluorescence probe whose fluorescence intensity is very sensitive to temperature in aqueous solutions. Both methods gave us similar values of *T*. Details of the *T* measurements are described in the Supporting Information (Fig. S6). At *I* = 15 kW/cm^2^, *ΔT* at the center of the irradiated area was evaluated to be *ΔT* = 41 K and hence *T* = 334 K. The irradiated area had a spatial *T* gradient with ∇*T* = −3.4 K/μm.

Considering these parameters, we evaluated the thermophoretic force *f*_t_ exerted on the DNA. As shown in Table 1, *f*_t_ increases as the size of the DNA increases. As previously described, *f*_t_ (>0) should act as a dispersing force, which transports DNA from the hotter (center of the irradiated area) to the colder area (outside the irradiated area)^34^. It is this thermophoretic force combined with the enhanced optical force due to plasmonic optical trapping that led to the formation of the DNA rings. Also, the origin of the dependence of the size of the ring on the size of the DNA can be ascribed to the DNA-size-dependent *f*_t_. Since *f*_t_ increases as the size of the DNA increases, larger DNA should be transported further from the center, resulting in the formation of a larger ring.

**Table 1:** Soret coefficient and thermophoretic force exerted on the DNA.

DNA (bp)	St [K^−1^]	f_t_ (fN)
φx DNA (5.4 kbp)	0.14^a^	2.2
λ-DNA (48.5 kbp)	2.1^b^	33
T4 DNA (165.6 kbp)	3.9^b^	61

^a^*S*_t_ of φx DNA (5.4 kbp) was assumed to be equal to that of plasmid DNA (5.6 kbp)^49^.

^b^*S*_t_ values were evaluated depending on the number of base pairs^38^.

The value of *f*_t_ for these DNA strands lies in the order of 10^−15^ ~10^−14^ N (several fN or several tens of fN). Obviously, these values are close to those of the trapping force *F*_t_ (0 ~ −50 fN, depending of the distance between the DNA and the nanostructure). This means that these opposite forces, attractive *F*_g_ and repulsive *f*_t_ for the plasmonic structure, compete with each other. When *F*_g_ and *f*_t_ are in balance, the DNA is trapped and immobilized forming separate rings. Thus this technique can be used as the basis of chromatographic separation.

Finally, we compare our technique with important research by Maeda *et al*.^50,51^. They prepared an aqueous solution containing a mixture of T4 DNA or RNA (0. 1 and 3 kbp) and polyethylene glycol (PEG, 1~5% in volume fraction). In the solution, PEG plays two important roles. First, it induces coil-globule phase transition of the T4 DNA. In the presence of PEG, random coil DNA with loose structures is converted to globule DNA with compact structures. This assists optical trapping, since it is very difficult to optically trap random coil DNA. Second, PEG can invert the direction of the thermophoresis for small objects involving DNA and RNA. Into the mixture solution, they focused a high power (200 mW maximum) near-infrared laser beam (λ = 1480 nm) corresponding to the order of MW/cm^2^ at the focal point. This induced a local temperature rise via vibrational excitation of the water, and created a huge temperature gradient (∇*T* = −0. 25 K/μm) around the focal point. Due to the presence of PEG, the T4 DNA and RNA were collected (trapped) and concentrated at the focal point by the inverted thermophoresis. For the solution with long RNA (3 kbp) and short RNA (0.1 kbp), both types of RNA were trapped with a separation between them. They observed a transient double-ring-pattern at the focal point by fluorescence microscopy. The origin of the separation was not the optical force (*F*_*g*_) but the RNA-size-dependent thermophoretic force. The effect of *F*_*g*_ would have been negligible in their case.

They successfully interpreted the phenomenon by constructing a novel theoretical model. Comparing that method with the plasmonic method presented here demonstrates several characteristics for the manipulation of DNA. First, our method does not require intense irradiation because the plasmons enhance *F*_g_ and electron-lattice relaxation efficiently generates ∇*T*. Second, our method does not require any chemical additives (such as PEG) because we get stable trapping of the DNA by the optical force. Third, we can immobilize the DNA on a solid substrate with a separate double-ring pattern. This is very favorable for the micro-analysis of DNA analytes. On the other hand, our method requires precisely-fabricated plasmonic nanostructures. These need to be fabricated by complicated chemical procedures taking a long time. Both our method and Maeda’s method are effective for DNA manipulation, and should be used complementarily according to the requirements of the DNA manipulation.

In conclusion, we demonstrated that three types of DNA with different sizes were optically trapped using plasmonic optical tweezers. DNA strands were permanently immobilized on the substrates creating micro-ring patterns. As the length of the DNA strand increased, the ring became larger. When we carried out trapping in solutions of mixed DNA, the DNA strands were immobilized creating double-ring patterns. In the double ring, the strands of DNA were separated; the shorter and longer strands forming the smaller and larger rings, respectively. We quantitatively interpreted this phenomenon in terms of the force balance between the plasmon-enhanced gradient force and the thermophoretic force. The results open future perspectives for separation and fixation of other biomolecules such as proteins by using this technique.

## Methods

As trapping targets, we used three kinds of DNA. φx174 DNA (5.4 kbp, abbreviated as φx DNA), lambda DNA (48.5 kbp, abbreviated as λ-DNA), and T4 GT7 DNA (165.6 kbp, abbreviated as T4 DNA). The DNA molecules were supplied by Nippon Gene. As fluorescent probes, YOYO-1 (λ_em_ = 510 nm), ethidium bromide (EtBr), and 4’,6-diamidino-2-phenylindole (DAPI, λ_em_ = 470 nm) were obtained from Invitrogen, Tokyo Kasei, and Wako, respectively, and used as received. A buffer solution (HEPES buffer solution, Wako, pH = 7.6) was used for the solvent. To suppress photobleaching of the fluorescent dyes, glucose (30 mmol/*l*), glucose oxidase (1 mg/m*l*) and catalase (1 mg/m*l*) were added to the buffer solution in the usual manner. The DNA molecules were stained with the fluorescent dyes by intercalation in the buffer solutions. The concentrations of the DNA and intercalating fluorescence dyes were adjusted to be 1.0 ~ 2.0 × 10^−6^ mol/L and 2.0 × 10^−6^ mol/L, respectively.

For the plasmonic substrate, we fabricated a gold nanopyramidal dimer array on a glass substrate by means of angular-resolved nanosphere lithography (AR-NSL)^52–54^. The plasmonic substrate has a broad extinction band around 800 nm (see Fig. S1), which is ascribed to a gap-mode plasmonic resonance. Resonant light irradiation around the wavelength results in an enhancement of the electromagnetic field (*E*) around the nanogaps by a factor of more than 5 × 10^3^ for (*E*/*E*_0_)^2^. The plasmonic nanostructure was brought into contact with the sample solution in a sample cell.

Details of our plasmonic optical trapping system combined with fluorescence microspectroscopy have been described elsewhere and in the Supporting Information (Fig. S1)^33,34,55^, and are briefly introduced here. We used a cw near-infrared (NIR) laser (λ = 808 nm) for LSP excitation and cw near-ultraviolet and visible lasers (λ = 375, 473 nm) for fluorescence excitation. These laser beams were co-axially introduced into a confocal optical microscope, and focused onto the sample cell. The trapping behavior was analyzed by monitoring the fluorescence of the samples.

## Supplementary information

Supplementary Information.
