# Supplementary material for: Plasmonic Manipulation of DNA using a Combination of Optical and Thermophoretic Forces: Separation of Different-Sized DNA from Mixture Solution
Source: Sci Rep. 2020 Feb 25;10:3349. doi: 10.1038/s41598-020-60165-5 (PMC7042363; doi:10.1038/s41598-020-60165-5)
Supplement: Supplementary file 1 — Supplementary Information. [file 41598_2020_60165_MOESM1_ESM.pdf]

Supplementary Information

Plasmonic Manipulation of DNA using a Combination of Optical and Thermophoretic Forces: Separation of Different-Sized DNA from Mixture Solution

Tatsuya Shoji[1,2], Kenta Itoh[1], Junki Saitoh[3], Noboru Kitamura[3], Takahiro Yoshii[3], Kei Murakoshi[3], Yuto Yamada[4], Tomohiro Yokoyama[4], Hajime Ishihara[4,5], and Yasuyuki Tsuboi[1,2]*

[1]*Division of Molecular Materials Science, Graduate School of Science, and*

[2]*The OCU Advanced Research Institute for Natural Science and Technology (OCARINA), Osaka City University, 3-3-138 Sugimoto, Sumiyoshi, Osaka 5558-8585, Japan.*

[3]*Department of Chemistry, Graduate School of Science, Hokkaido University, Sapporo, Hokkaido 060-0810, Japan.*

[4]*Division of Materials Physics, Graduate School of Engineering Science, Osaka University, 1-3 Machikaneyama, Toyonaka, Osaka 560-8531, Japan.*

[5]*Department of Physics and Electronics, Graduate School of Engineering, Osaka Prefecture University, 1-1, Gakuen-cho, Nakaku, Sakai, Osaka 599-8531, Japan*

Plasmonic nanostructures

We fabricated a a gold nanopyramidal dimer array on a glass substrate by means of angular-resolved nanosphere lithography (AR-NSL).[1–3] Representative polarized extinction spectra shows Fig. S1(a). The plasmonic substrate has a broad extinction band around 800 nm, which is ascribed to a gap-mode plasmonic resonance of gold nanopyramids (Fig. S1(b)[4]). We have already discussed the enhancement effect of the plasmonic nanostructures by means of theoretical calculation (discrete dipole approximation) in elsewhere[4–6]. The electric field of resonant light (λ = 808 nm) is strongly enhanced at the nanogaps between the nanopyramids.

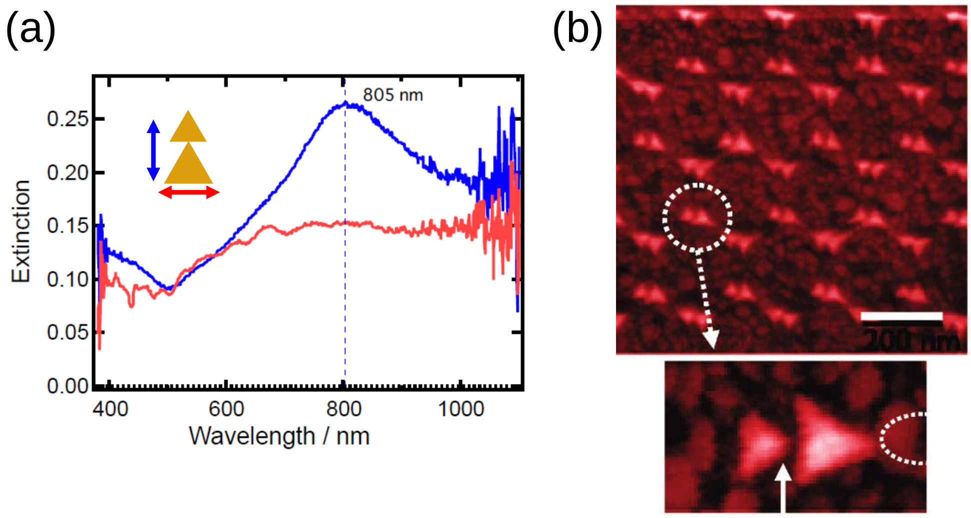

Fig. S1

(a) Representative polarized extinction spectra from an AR-NSL substrate. The polarized angles of incident light were parallel (blue) and perpendicular (red) to the long axis of the dimers, respectively.

(b) A SEM image of the AR-NSL substrate. Scale bar is 200 nm. Reprinted with permission from [4]. Copyright 2010 American Chemical Society.

Optical setup for plasmonic optical tweezers combined with confocal fluorescence microspectrosocpy

We have already described the detail of optical setup for plasmonic optical tweezers combined with confocal fluorescence microspectroscopy in elsewhere[5,7,8]. A continuous wave (cw) near-infrared (NIR) diode laser beam (wavelength λ = 808 nm) was used for a plasmon excitation light source. A cw near-ultraviolet (λ = 375 nm) and visible lasers (λ = 473 nm) were used for fluorescence excitation light sources. These laser beams were coaxially introduced into an inverted confocal optical microscope (Nikon, ECLIPSE TI-U). Using oil-immersion objective lens (Nikon, x 100 magnification, *N.A.* = 1.40), a cw NIR laser beam was loosely focused on plasmonic surfaces contacted with DNA sample solutions. Fluorescence signals were detected by a CCD camera (Andor Tec.) equipped with a polychromator (Acton research, Spectra Pro 2300i, grating: 600 lines/ mm) for spectral measurements. For bright-field and fluorescence microscopic observations, we used a CMOS color camera and optical filters.

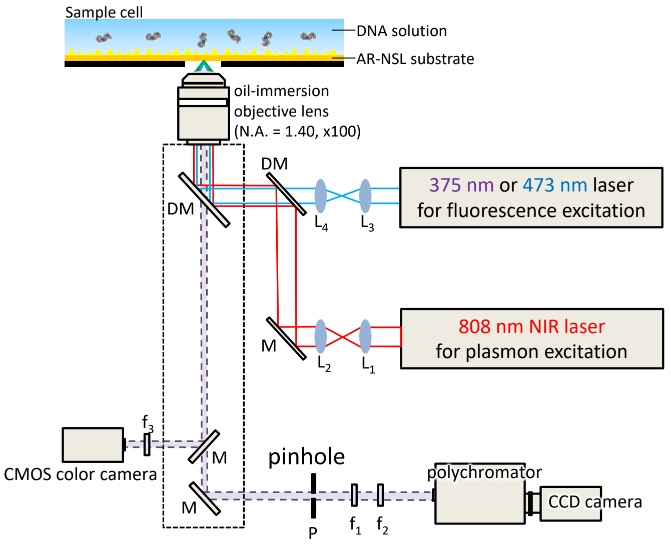

Fig. S2

A schematic diagram of optical setup. M: mirror, L: lens, DM: dichroic mirror, f: optical filter.

DNA micro-ring formation by changing excitation intensity and irradiation area

Fig. S3 shows bright-field micrographs during plasmonic optical trapping of λ-DNA by changing plasmon excitation intensity. We successfully formed a DNA micro-ring on a plasmon substrate in a intensity range of 10 – 20 kW/cm^2. Below 10 kW/cm^2, we have never observed any sign during plasmon excitation. Over 20 kW/cm^2, a micro-bubble formation was observed at the irradiation area by a local photothermal effect of the excited gold nanostructures. The diameters of the micro-rings (D_{ring}) were evaluated to be 7.5 μm at 13 kW/cm^2, 8.3 μm at 15 kW/cm^2, 9.1 μm at 17 kW/cm^2, and 9.8 μm at 20 kW/cm^2. These results indicated that the micro-ring size was controllable by changing plasmon excitation intensity.

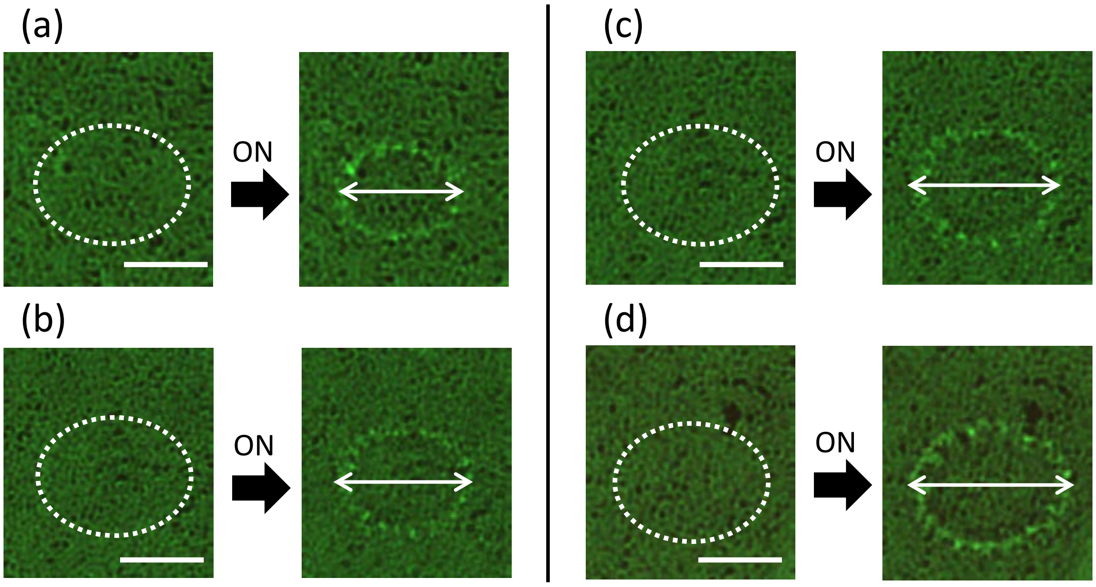

Fig. S3

Bright-field micrographs of plasmonic optical trapping of λ-DNA by changing excitation intensity: (a) 13, (b) 15, (c) 17 and (d) 20 kW/cm^2. Plasmon excitation area is expressed as a white circle. Scale bar are 5 μm. DNA concentrations are 2.0×10^{-6} mol/L. D_{ring}: (a) 7.5, (b) 8.3, (c) 9.1, (d) 9.8 μm.

The micro-ring size was also controllable by changing the size of irradiation area. Fig. S4 shows bright-field micrographs during plasmonic optical trapping of λ-DNA by changing the size of irradiation area. D_{ring} of the micro-ring became larger with increasing the irradiation area: (a) 4.6 μm (26 μm^2), 6.5 μm (35 μm^2), 7.9 μm (45 μm^2), and 8.3 mm (51 μm^2).

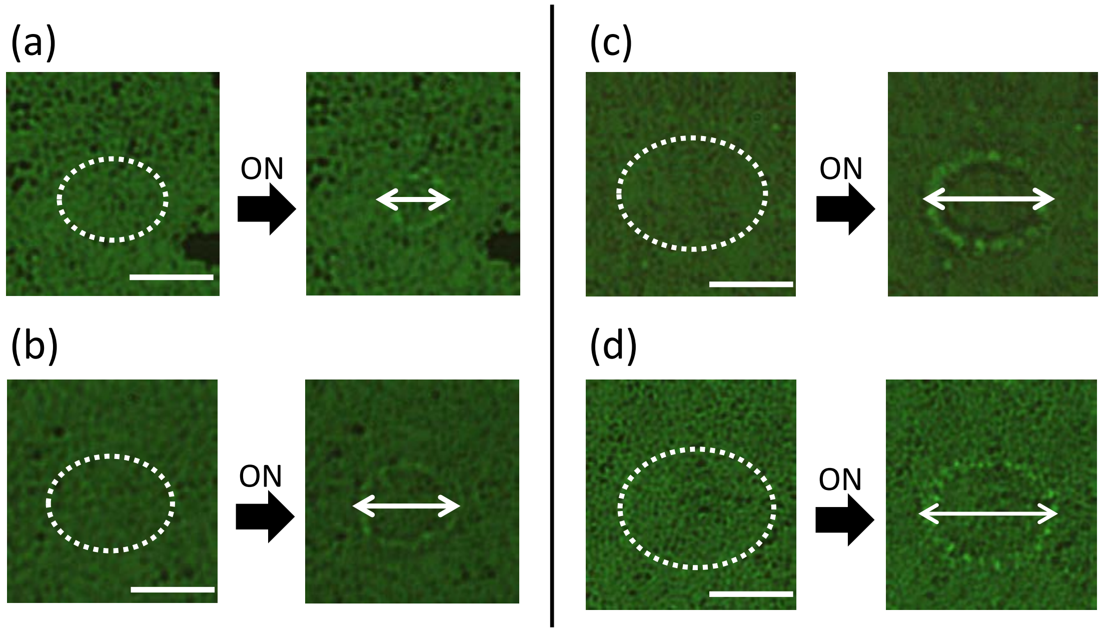

Fig. S4

Bright-field micrographs of plasmonic optical trapping of λ-DNA by changing irradiation area: (a) 26, (b) 35, (c) 45 and (d) 51 μm^2. Plasmon excitation intensity: 15 kW/cm^2. Scale bar are 5 μm. DNA concentrations are 2.0×10^{-6} mol/L. D_{ring}: (a) 4.6, (b) 6.5, (c) 7.9, (d) 8.3 μm.

Spatially-resolved fluorescence spectra of a micro-ring of T4 DNA and φx DNA (Fig.2)

Fig. S5 shows spatially-resolved fluorescence spectra for DNA micro-rings. These micro-rings were made of φx DNA (a) and T4 DNA (b). The details of optical micrographs during the micro-ring formation were described in the manuscript. φx DNA was stained with red fluorescence molecular probes (ethidium bromide, EtBr), while T4 DNA was stained with blue fluorescence molecular probes (4’6-diamidino-2-phenylindole, DAPI). At the center of the irradiation area and at an outer-side of the ring, fluorescence was hardly detected. On the other hand, just at the ring, we clearly observed fluorescence of EtBr and DAPI, respectively. These results indicated that each rings were identified to the corresponding DNAs.

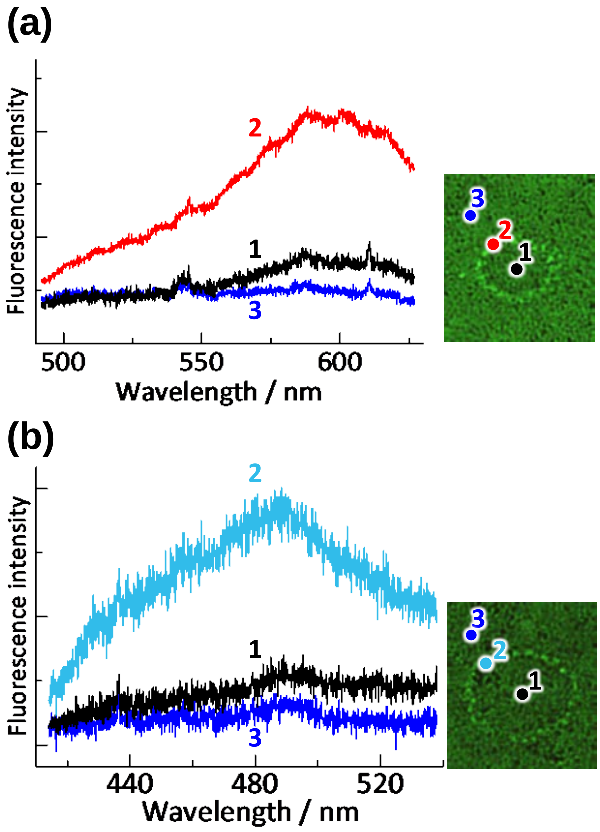

Fig. S5

Spatially-resolved fluorescence spectra for the DNA micro-ring, (a) φx DNA labelled with EtBr, (b) T4 DNA labelled with DAPI: (1) at the center of the irradiation area, (2) just at the ring, (3) at an outer-side of the ring.

Experimental determination of temperature elevation during plasmon excitation

We determined temperature elevation to obtain fluorescence spectra of a thermoresponsive fluorescence dye (2’,7’-bis(2-carboxyethyl)-5-(6)-carboxyfluorescein, BCECF) located on a plasmonic substrate during plasmon excitation. BCECF has fluorescence intensity sensitive to temperature. The fluorescence intensity of the dye decreased by 7.8 % when temperature increased by 10 K. Using this molecular probe, we precisely determined temperature elevation during plasmon excitation for obtaining temperature gradient around the plasmon excitation area. Fig. S6(a) shows fluorescence spectra of BCECF at the center of plasmon excitation area. Increasing plasmon excitation intensity, the fluorescence intensity decreased. We determined temperature elevation ΔT from the room temperature (Fig. S6(b)) based on the fluorescence spectra (Fig. S6(a)). At 15 kW/cm^2, we determined ΔT to be 41 K. By using this technique for temperature gradient around the plasmon excitation area, we also obtained the spatially resolved fluorescence spectra during plasmon excitation at each distance r from the center of the area to the outer-side of the area (Fig. S6(c)). Fig. S6(d) shows temperature elevation ΔT at distance r by changing plasmon excitation intensity. The slopes of these fitting curves correspond to the spatial gradient of temperature. We determined a spatial temperature gradient to be $\nabla \mathrm{T} = -3.4$ K/μm at 15 kW/cm^2.

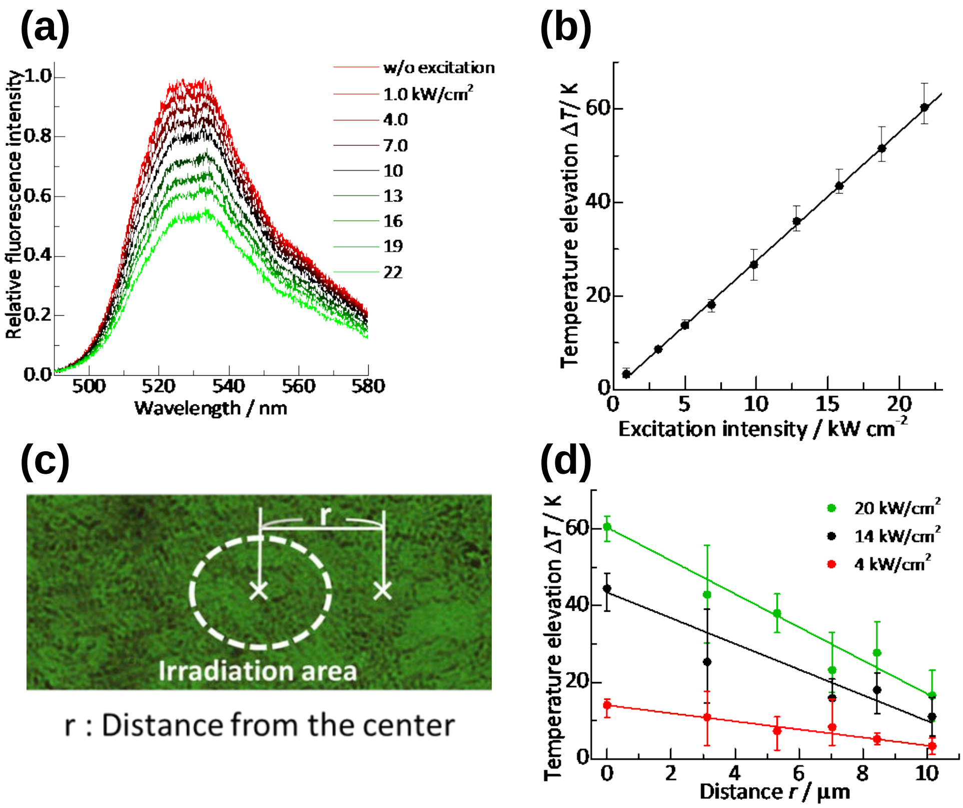

Fig. S6

(a) Fluorescence spectra of BCECF during plasmon excitation (1.0 – 22 kW/cm^2) together with that without the excitation (red). (b) Plasmon excitation intensity dependence of temperature elevation from room temperature obtained from (a). (c) A bright-field micrograph of plasmon nanostructures. White circle is expressed as plasmon excitation area. (d) Temperature elevation along the distance from the center of plasmon excitation area to an outer-side of the area.

References

1. Haynes, C. L., McFarland, A. D., Smith, M. T., Hulteen, J. C. & Van Duyne, R. P. Angle-resolved nanosphere lithography: Manipulation of nanoparticle size, shape, and interparticle spacing. *J. Phys. Chem. B* **106**, 1898–1902 (2002).
2. Takase, M. *et al.* Selection-rule breakdown in plasmon-induced electronic excitation of an isolated single-walled carbon nanotube. *Nat. Photonics* **7**, 550–554 (2013).
3. Shoji, T. & Tsuboi, Y. Plasmonic Optical Tweezers toward Molecular Manipulation: Tailoring Plasmonic Nanostructure, Light Source, and Resonant Trapping. *J. Phys. Chem. Lett.* **5**, 2957–2967 (2014).
4. Tsuboi, Y. *et al.* Optical Trapping of Quantum Dots Based on Gap-Mode-Excitation of Localized Surface Plasmon. *J. Phys. Chem. Lett.* **1**, 2327–2333 (2010).
5. Shoji, T. *et al.* Plasmon-Based Optical Trapping of Polymer Nano-Spheres as Explored by Confocal Fluorescence Microspectroscopy: A Possible Mechanism of a Resonant Excitation Effect. *Jpn. J. Appl. Phys.* **51**, 092001 (2012).
6. Toshimitsu, M. *et al.* Metallic-Nanostructure-Enhanced Optical Trapping of Flexible Polymer Chains in Aqueous Solution As Revealed by Confocal Fluorescence Microspectroscopy. *J. Phys. Chem. C* **116**, 14610–14618 (2012).
7. Shoji, T. *et al.* Highly Sensitive Detection of Organic Molecules on the Basis of a Poly(N -isopropylacrylamide) Microassembly Formed by Plasmonic Optical Trapping. *Anal. Chem.* **89**, 532–537 (2017).
8. Shoji, T. *et al.* Permanent Fixing or Reversible Trapping and Release of DNA Micropatterns on a Gold Nanostructure Using Continuous-Wave or Femtosecond-Pulsed Near-Infrared Laser Light. *J. Am. Chem. Soc.* **135**, 6643–6648 (2013).
